# Anti-MDA5 Antibody-Positive Clinically Amyopathic Dermatomyositis Associated With Multiple Heterologous Carcinomas: A Case Report

**DOI:** 10.7759/cureus.54660

**Published:** 2024-02-21

**Authors:** Yoshio Nakano, Koji Nishida, Norio Okamoto, Iwao Gohma, Yumiko Yasuhara

**Affiliations:** 1 Respiratory Medicine, Sakai City Medical Center, Osaka, JPN; 2 Pathology, Sakai City Medical Center, Osaka, JPN

**Keywords:** heterochronous, cancer, interstitial lung disease, anti-mda5 antibody, dermatomyositis

## Abstract

Dermatomyositis (DM), an autoimmune disorder, is linked to increased malignancy risk. A 53-year-old man with anti-melanoma differentiation-associated gene 5 (MDA5)-positive clinically amyopathic dermatomyositis (CADM) and rapidly progressing interstitial lung disease (RP-ILD) developed heterochronous gastric and colorectal cancers. Early endoscopic screenings led to successful curative resections, preventing recurrence. Despite low cancer incidence assumptions in patients with anti-MDA5-positive RP-ILD, this case advocates for reevaluation and periodic cancer screenings to enhance management, considering the improved survival with intensive therapy. Vigilance for multiple carcinomas at various time points is vital in CADM management.

## Introduction

Dermatomyositis (DM) is an autoimmune disorder characterized by the inflammation of the skin and the muscles. Patients with DM are at an elevated risk of developing malignancies [1].

The presence of anti-melanoma differentiation-associated gene 5 (MDA5) antibodies in clinically amyopathic dermatomyositis (CADM) is frequently associated with rapidly progressing interstitial lung disease (RP-ILD) and increased mortality rates [2]. Current therapeutic strategies emphasize early and aggressive immunotherapy, with studies indicating improvements in mortality rates when initiated at the early stages of the disease [3]. Approximately 20% of patients with DM demonstrate positivity for the anti-MDA5 antibody, underscoring the clinical significance of this particular autoantibody [4].

Research has shown that the anti-TIF1-γ antibody is linked to cancer in patients with DM, whereas the anti-MDA5 antibody has not been found to be associated with cancer [5]. Furthermore, the long-term risk of cancer in dermatomyositis, particularly in cases of anti-MDA5 antibody positivity and RP-ILD, remains inadequately explored. The extant literature points to a potential reduction in the risk of cancer over time subsequent to a DM diagnosis [6]. Although there are scattered reports of heterochronous multiple carcinomas in DM [7,8], there have been no reports of heterochronous multiple cancers in patients with RP-ILD who are positive for anti-MDA5 antibody over a long period.

The survival rate for patients with RP-ILD who are positive for the anti-MDA5 antibody has shown improvement, and the incidence of long-term survivors is anticipated to rise. Consequently, vigilance for the emergence of heterochronous multiple carcinomas is warranted.

In this instance, a patient with RP-ILD who was positive for the anti-MDA5 antibody developed multiple heterochronous cancers over an extended duration. Fortunately, due to early detection facilitated by regular upper and lower endoscopy, both cancers were treatable and resectable.

## Case presentation

**Figure 1:**
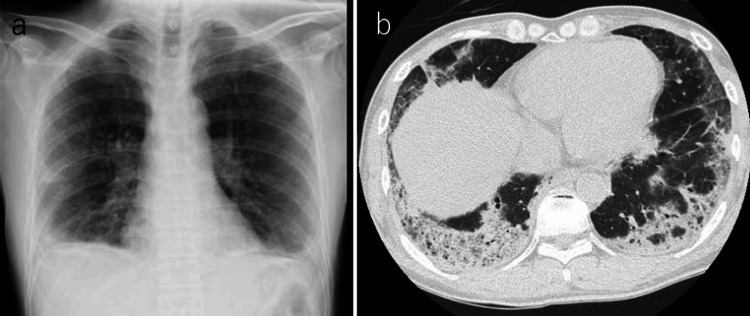
Chest imaging upon admission. a: Chest X-ray revealing a frosted shadow with decreased volume in the bilateral lower lung fields. b: Chest CT image displaying infiltrative shadows and frosted patterns predominantly observed in the outer lower lobes on both sides.

The patient, a 53-year-old man, noted the onset of a facial skin rash and fever approximately two weeks prior to consultation. One week before his visit, symptoms of weakness and malaise manifested, and approximately four days before the visit, he experienced respiratory distress. He was referred to our hospital by a local physician on suspicion of interstitial pneumonia. Upon examination, his vital signs were as follows: clear consciousness, blood pressure at 128/75 mmHg, pulse rate at 80 beats/min, saturation of peripheral oxygen (SpO2) at 92% under 3 L/min of oxygen supplementation, respiratory rate at 30 breaths/min, and body temperature at 37.9°C. A physical examination revealed fine crackles in both lung fields, a heliotrope rash on his face, mechanic's hand signs, and Gottron's sign on his hands, without evident muscle weakness but with palpable pain in the thigh muscles. Chest CT scanning revealed infiltrative shadows with volume loss predominantly in the peripheral areas of the bilateral lower lungs (Figure 1).

Blood tests indicated elevated levels of creatine kinase (CK), aldolase, and carcinoembryonic antigen (CEA) (Table 1).

**Table 1:** Laboratory test results on admission to our hospital.

Laboratory test	Results	Normal range
WBC count	5140	3300-8600 10^4^/μL
Hemoglobin	12	13.7-16.8 g/dL
Platelet count	43.4	15.8-34.8 10^4^/μL
Aspartate aminotransferase	107	13-33 U/L
Alanine aminotransferase	61	8-42 U/L
Lactate dehydrogenase	548	119-229 U/L
Creatine kinase	948	62-287 U/L
Aldolase	18.7	2.7-7.5 U/L
Creatinine	0.56	0.53-1.02 mg/dL
C-reactive protein	1.6	0-0.3 mg/dL
Ferritin	598	50-200 ng/mL
KL-6	1244	105-401U/mL
Anti-MDA5 antibody	>150	0-32 Index
Anti-SSA antibody	65.5	0-6.9 U/mL
Anti-TIF1γ antibody	<5	0-32 Index
Carcinoembryonic antigen	59.8	0-5 ng/mL

Given these findings, a diagnosis of anti-MDA5 antibody-positive CADM with RP-ILD was suspected, and immediate treatment with a steroid pulse (methylprednisolone 1000 mg/d for three days), IV cyclophosphamide pulse (IVCY, 500 mg/d), and cyclosporine (CyA 150 mg/d) was initiated. By the third day, oxygenation had deteriorated to an SpO2 of 92%, requiring support with a high-flow nasal cannula at a fraction of inspired oxygen (FiO2) of 40% and a gas flow of 40 L/min. Concurrently, he tested positive for anti-MDA5 antibodies, confirming the diagnosis of anti-MDA5 antibody-positive CADM with RP-ILD, while tests for anti-TIF1γ antibody returned negative. Subsequently, the patient's oxygenation levels gradually improved, allowing for his discharge on the 39th day with long-term oxygen therapy (LTOT). The initial upper and lower endoscopies, conducted on the 24th day, revealed no evident tumors.

**Figure 2:**
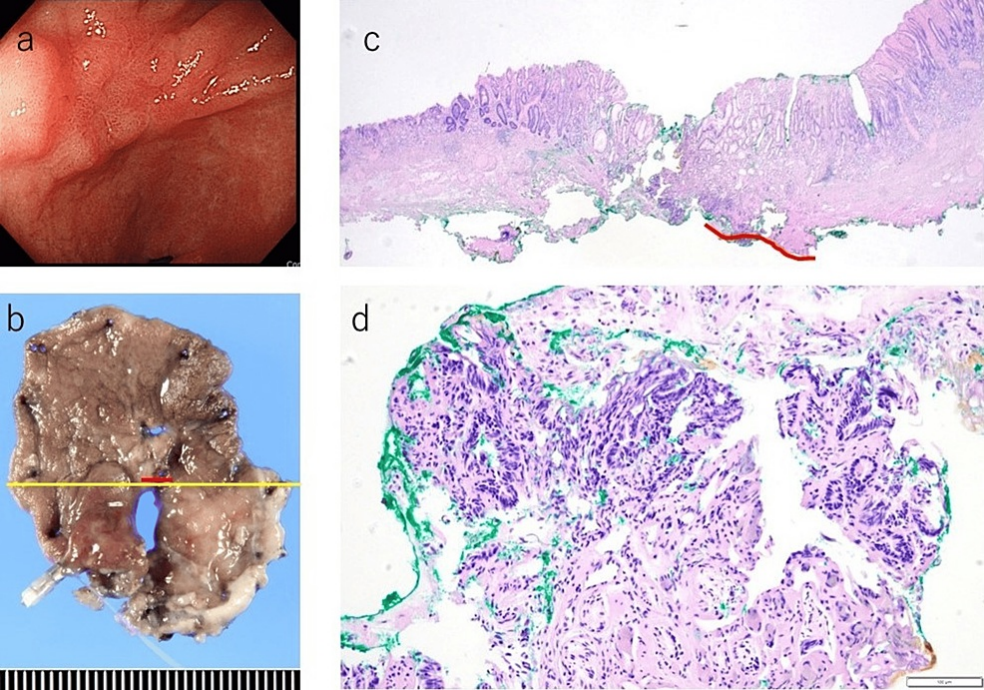
At one year and eight months post-disease onset, upper and lower endoscopies unveil early gastric cancer. a: An 8-mm-sized erythematous depression can be noted in the vestibular fontanel. b: Resection specimen from gastric cancer surgery. c: Diagnosis of highly differentiated ductal adenocarcinoma is established based on irregular ducts exhibiting nuclear enlargement and pseudo-stratification. H&E staining; original magnification ×20. d: Original magnification ×200.

The CEA levels gradually decreased and normalized approximately one year and 10 months post-diagnosis. The interstitial pneumonia resolved, and anti-MDA-5 antibody levels normalized within about three years. At one year and eight months following disease onset, upper and lower endoscopies detected early-stage gastric cancer. At one year and 10 months post-diagnosis, an endoscopic submucosal dissection (ESD) was performed, with subsequent follow-up examinations showing no recurrence of gastric cancer. Pathological analysis identified the tumor as a well-differentiated adenocarcinoma measuring 3.1 × 2 mm, confined to the mucosa, leading to a diagnosis of stage IA gastric cancer (pT1aN0M0) (Figure 2).

**Figure 3:**
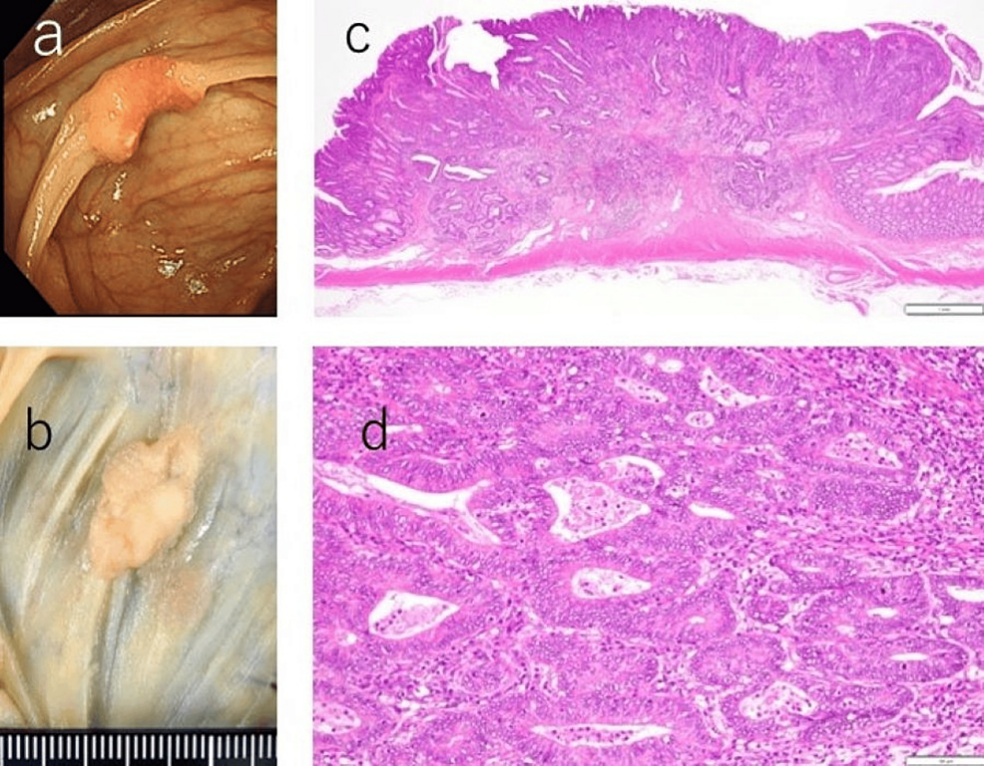
At two years and 10 months post-disease onset, lower gastrointestinal endoscopy reveals a type 2 tumor. a: An approximately 20 mm tumor is located in the cecum, positioned distally to the Bauhin valve. b: A 21×12 mm large type 2+0-IIa tumor is observed in the cecum, with an 8×8 mm 0-IIa lesion in the vicinity. c: Proliferation of atypical cells displaying irregular glandular duct formation is observed, infiltrating the submucosa. H&E staining; original magnification ×20. d: Original magnification ×200.

Colorectal cancer (adenocarcinoma of the cecum) was discovered via lower gastrointestinal endoscopy two years and 10 months after the initial disease presentation. An open ileal resection was conducted. Pathological evaluation of the resected specimen revealed two tumors, each diagnosed as colorectal cancer. The first lesion was identified as a tubular adenocarcinoma, classified from well to moderately differentiated (tub1>tub2), with dimensions of 21×12 mm, and classified as type 2+0-IIa, stage I (pT1b(SM, 3000 um)N0M0). The second lesion was a well-differentiated tubular adenocarcinoma (tub1), measuring 8×8 mm, classified as type 0-IIa, stage 0 (pTis(M)N0M0) (Figure 3).

Despite the complication of tumors, there was no increase in CEA levels and deterioration of interstitial pneumonia or dermatological symptoms. As of six years and four months post-disease onset, there has been no recurrence of the tumor.

## Discussion

In this case, heterochronous multiple cancers were documented over a prolonged period following the stabilization of RP-ILD in a patient positive for anti-MDA-5 antibodies. Both gastric and colorectal cancers were successfully resected, with no recurrence noted to date. The cornerstone of this favorable outcome was the early detection enabled by consistent upper and lower endoscopy screenings.

Previous research has identified a heightened risk of cancer complications in patients with dermatomyositis positive for anti-Tif1γ antibodies, whereas anti-MDA-5 antibodies were not traditionally associated with an increased cancer risk [5]. There have been limited reports of cancer occurrences in patients with anti-MDA-5 antibody-positive RP-ILD [9, 10]. Moreover, no instances of heterochronous multiple carcinomas in patients with anti-MDA-5 antibody-positive RP-ILD have been reported following long-term disease stabilization. A review highlights the clinical significance of ADM due to its correlation with an elevated risk of rapidly progressing fatal interstitial lung disease (ILD) and the potential for internal malignancy [11]. Anti-MDA5 antibody-positive CADM is also potentially linked to cancer. This case illustrates that patients with acute progressive ILD who test positive for anti-MDA-5 antibodies may also be at risk of developing heterochronous multiple cancers over an extended duration.

RP-ILD associated with positive anti-MDA-5 antibodies is characterized by high mortality rates. However, early intervention with potent steroids and immunosuppressive medications has been documented to enhance survival outcomes [3]. In this specific case, the patient, presenting with RP-ILD and characteristic dermatological manifestations, had his life saved through the immediate administration of steroid pulses, cyclosporine, and cyclophosphamide upon admission, predicated on the initial diagnosis of DM with positive anti-MDA-5 antibodies. The survival prospects for individuals with RP-ILD positive for the anti-MDA5 antibody have been showing improvement with aggressive therapy, and as such, the prevalence of long-term survivors is anticipated to rise. This underscores the importance of vigilance for the potential development of heterochronous multiple carcinomas.

Although cancer screening is recommended at the diagnosis of DM, the efficacy of subsequent cancer screenings for patients with an established diagnosis of DM is not elucidated. There have been reports that the exacerbation of skin symptoms and an increase in anti-TIF1-γ antibody levels are indicative of cancer, highlighting the importance of monitoring these indicators for early cancer detection in patients with dermatomyositis [7]. In this case, cancer was diagnosed exclusively through routine upper and lower endoscopy, due to the lack of discernible elevations in tumor markers, exacerbation of cutaneous symptoms, or signs of interstitial pneumonia. This case suggests that periodic screening for cancer may be useful even in rapidly progressive interstitial pneumonia with positive anti-MDA-5 antibody, which is considered to have a low incidence of cancer.

Muscles affected by myositis exhibit elevated levels of autoantigens, establishing a connection between malignancy and inflammatory myopathy through the shared expression of autoantigens in both cancerous and muscle tissues. This suggests that immune responses directed at tumors may also inflict damage on muscle tissue in cases of DM or polymyositis (PM) [12]. Recent investigations underscore a paraneoplastic association between DM and cancer, with approximately 24% of DM incidents linked to malignancies. This connection suggests an immunological foundation, characterized by distinct immunophenotypic profiles, including a predominance of CD4+ T cells and B cells in DM, alongside a decrease in regulatory T cells. Such profiles indicate a maladaptive immune response to self-antigens and a potential interaction with antigens expressed by cancer cells [13]. These observations highlight the importance of immune dysregulation in the etiology of DM and its significance as an indicator of concealed malignancies.

CEA is frequently used as a tumor marker and is known to be elevated in a variety of gastrointestinal cancers, among others. Although the precise mechanism remains elusive, reports of heightened CEA levels in patients with CADM who lack concurrent tumors have identified this as a significant marker of RP-ILD and a predictor of poor outcomes in these patients [14]. In the case described, the patient presented with elevated CEA levels without any detectable tumor initially. Nonetheless, over an extended period, the patient developed multiple heterologous cancers, suggesting a potential immunological link between CADM and malignancies.

This case underscores the potential for heterochronous multiple cancers in anti-MDA-5 antibody-positive rapidly progressive interstitial pneumonia, challenging perceptions of low cancer incidence. Successful outcomes, achieved through early detection via regular endoscopy screenings, emphasize the importance of vigilant monitoring.

As the survival rate improves with intense therapy, long-term cases are expected to rise, necessitating continued attention to heterochronous multiple carcinomas. Even in conditions deemed low risk, periodic cancer screenings emerge as a potential tool for better management.

## Conclusions

In this case study, we presented a patient with anti-MDA5 antibody-positive CADM who developed multiple cancers, underscoring the necessity for rigorous cancer screening in such patients. Our findings suggest reevaluating cancer risk in CADM, particularly with anti-MDA5 antibodies, advocating for comprehensive cancer surveillance. This approach may enhance early detection and treatment of cancers in this vulnerable group, potentially improving patient outcomes. Our research indicates a need for further investigation into the link between CADM, anti-MDA5 antibodies, and cancer risk, pointing toward more proactive screening protocols.
